# Unique challenges for health equity in Latin America: situating the roles of priority-setting and judicial enforcement

**DOI:** 10.1186/s12939-019-1005-3

**Published:** 2019-07-04

**Authors:** Alicia Ely Yamin, Andrés Pichon-Riviere, Paola Bergallo

**Affiliations:** 1 Global Health Education and Learning Incubator at Harvard University, Harvard University, 104 Mt. Auburn St, 3rd floor, Cambridge, MA 02138 USA; 2 Institute for Clinical Effectiveness and Health Policy (IECS), University of Buenos Aires, Dr. Emilio Ravignani 2024, C1414 CPV Buenos Aires, Argentina; 3 Universidad Torcuato di Tella, Av. Pres. Figueroa Alcorta 7350, C1428 CABA Buenos Aires, Argentina

**Keywords:** Latin America, Health systems, Judicialization, Priority-setting, Right to health, Democratic institutions

## Abstract

Overcoming continuing polarization regarding judicial enforcement of health rights in Latin America requires clarifying divergent normative and political premises, addressing the lack of reliable empirical data, and establishing the conditions for fruitful inter-sectoral, inter-disciplinary dialogue.

## Background

Although an extremely diverse region, Latin America poses a series of challenges for achieving equity in health. First, both overall and within many individual countries high degrees of socio-economic inequality are refracted along racial, gender and ethnic lines, according to distinct demographic configurations. Health is an acute reflection of overall patterns of inequality and discrimination and, as elsewhere, social determinants contribute more to patterns and burdens of disease than medical care in the region [1]. The region is also deeply influenced by political determinants of health, defined by the Lancet-Oslo Commission in 2014 as the “norms, policies, and practices that arise from transnational interaction” [2]. For example, Argentina faces International Monetary Fund prescriptions, including austerity policies, which affect population health through multiple pathways.

Second, Latin America suffers from a generalized tendency toward both political and regulatory dysfunction. The former impairs the ability to collectively arrive at democratically justified decisions regarding health in particular, and social policy more broadly. The latter means that issues often addressed through administrative rule-making in other contexts present gaps or “gray zones,” and regulatory oversight is weak. Both political capture and polarization, and the lack of robust and effective regulation of public and private actors have particularly dramatic—and often gendered—impacts on health across the region.

Third, Latin America is characterized by fragmented, and highly medicalized health systems, which affects everything from financing to priority-setting to organization and delivery of care. As the burden of disease due to non-communicable diseases has grown as a proportion of the total disease burden, the medicalization of health systems has accelerated. Fragmentation as well as inequities can also be exacerbated in federalist systems where health provision, as well as social policy that affects health, is decentralized [3], such as Argentina.

Finally, many countries in the region have recognized the right to health autonomously in their constitution or by incorporation through international treaties, or both, and others have interpreted the right to life in ways that include aspects of health and health care. In turn, in these countries, individual protection writ actions (*amparos, tutelas*) can be used to obtain health–related entitlements from courts with relative ease. Some scholars and policy-makers to argue that judicial enforcement of health rights undermines equity as well as systematic efforts to achieve Universal Health Coverage (UHC) in accordance with the Sustainable Development Goals. In a 2017 comment in the *Lancet,* a multi-disciplinary group of authors debunked the blanket assumption of a conflict between judicialization and priority-setting [4]. Nevertheless, in Latin America, where individual protection writ actions for entitlements have run into the hundreds of thousands in some countries, these debates persist. In our view, overcoming continuing polarization requires clarifying divergent normative and political premises, addressing the lack of reliable empirical data, and establishing the conditions for fruitful inter-sectoral, inter-disciplinary dialogue.

### Points of departure: health systems, priority-setting, and the role of courts

Our starting point is, first, that if health is understood as a right, health systems must be understood as democratic social institutions that cannot be divorced from the quality and texture of the democracies in which they exist. A health system organized around the right to health is analogous to a justice system organized around the right to a fair trial, or an electoral system organized around the right of political participation [5].

Second, all rights require resources and have distributional consequences. Some impacts are more visible than others, due to historic trajectories and financing employed in the institutional arrangements that support the right (e.g. taxes versus employment-based or social insurance). All rights also have collective and individual dimensions. For example, the right of due process requires a fair justice system (financed generally through taxes) but also calls for rules to be applied fairly in specific cases. Similarly, the right to health requires a fairly financed and just set of institutions and priority-setting processes by which the contours of an enforceable entitlement are defined. At the same time, individuals have both freedoms and entitlements within the system.

We need not reflexively accept that a health budget is fair at any given moment to note that *no country in the world meets all health needs*. Thus, the pressing question, as Norman Daniels has written, is how can we meet them fairly? [6] There is now a broad consensus that, a fair and legitimate process should be, at a minimum: (1) transparent and accessible to the public; (2) based on reasons relevant to advancing population health (i.e. not on religious arguments); (3) revisable in light of new evidence and arguments that may apply to a specific case, and (4) subject to regulation and enforcement [6]. Further, increasingly, health scholars and ethicists, argue that meaningful citizen participation is necessary for democratic legitimacy [7]. Moreover, priority-setting must be institutionalized as multiple conditions change over time, and new evidence and interventions become available.

By contrast, implicit priority-setting, which is widely practiced in the region, relies on waiting lines, prices, shortages, bureaucratic delays, etc., to ration access to care [8]. Implicit priority-setting, which is inherently untransparent, may be more politically palatable in the short term. However, it is neither just (the well-off have better access; it fosters substantial inequities); nor does it enhance the legitimacy of the health system as a democratic institution in the long term. A WHO Task Force on Fair Choices on the Path to UHC noted the ethical imperative of explicit priority-setting [9]. A Policy Forum on Health Technology Assessments in the region, held in 2018, came to the same conclusion [10].

Finally, just as with other fundamental social institutions in a democracy, as guardians of our constitutional values, courts have an inherent role to play in ensuring that the health system reflects those shared norms of dignity and equality, whether addressing individual entitlements or collective actions [11]. High courts in Latin America have been able to establish principles and criteria relating to the functioning of a democratic health system, including: obligations of comprehensive and continuous care; regulation of private providers and insurers; periodic processes relating to determination of benefits; and rights to information (related to care as well as social determinants of health). In some instances, judgments have catalyzed important political action, by both the executive and legislature, such as a new statutory law on health in Colombia, or further regulation of pharmaceuticals, sugary beverages, alcohol and tobacco [5].

### What we know about the categories of individual litigation and judicial enforcement of health rights

Judicialization is complex and arises in various configurations across different countries; broad-brush-assessments are likely to be misleading or inaccurate. Nonetheless, although there are cases that combine aspects of each category and there are overlaps, it is useful to distinguish between three general types of litigation for individual entitlements. First, a significant portion of health rights litigation in the region relates to the enforcement of health entitlements to which health system users should already have access to but do not due to administrative failures and other obstacles in practice. This type of litigation often responds to regulatory compliance gaps. It is neither inconsistent with systematic priority-setting, nor should it be characterized as judicial activism. However, it may exacerbate inequities if the compliance gaps are not closed.

A second pattern of health rights litigation involves entitlements that fall into gray areas. Patients may litigate due to a lack of clarity regarding what is included in a health package (e.g. a prosthetic as well as the actual surgery for joint replacement, etc.). Others may litigate because they are denied a good or service without justification. The first set of cases point to a regulatory failure in the system; the latter set of cases may also point to regulatory failure, or to lack of transparency in the priority-setting process. In both instances, purposive judicial enforcement should incentivize more clarity and improved priority-setting processes. However, just as in compliance gap cases, in practice judicial enforcement of such entitlements can function more as an “escape valve” perpetuating the regulatory dysfunction in the system.

A third set of cases involves entitlements—especially, but not exclusively, costly medicines—that are expressly excluded from health schemes. Insofar as the exclusion is based upon both the application of legitimate criteria (e.g., lack of clinical effectiveness; lack of cost-effectiveness; weak evidence) *within a legitimate priority-setting process*, the ad hoc granting of health entitlements by courts to those who have access is not conducive to advancing equity across geographic regions or fragmented health systems [12].

### We need to know more about the dynamics of priority setting and judicialization to evaluate impacts and respond effectively

To understand the impacts on equity, as well as greater oversight or other social values we care about, we need to understand not just what is being litigated but who is litigating and what the direct and indirect impacts are. That is: (1) who are the claimants (class, gender, race, and other indicators of social status; geographic regions)?; (2) what are the success rates for different kinds of claims?; and (3) what are the direct and indirect impacts of cases [13], including how the costs are borne? For example, repeated litigation may lead to revising criteria for mandatory insurance schemes. However, clusters of cases may become “routinized” [14] and produce systemic transaction costs, as has occurred in Argentina. Further, universalization may be ethically justified, drive the price of medication or a service down, and promote equity; however, it may also respond to disproportionate political clout of a given patient community, or pharmaceutical industry advocacy.

In short, extrapolating conclusions at any given time or across different countries is likely to be fallacious without more granular information. Just as transparent data is necessary in a justice system, an electoral system or an education system to determine efficiency and fairness, so too do we need urgently better and more accessible information to enable a more accurate assessment of the dynamics between priority-setting processes, regulatory oversight and judicialization [15]. Ideally, such information would be presented in transparent and accessible format as close to real-time as possible, which permits comparative analyses across time and both sub-national and national contexts.

## Conclusions

Advancing health —and social—equity in the region requires addressing the broader factors that promote systematic exclusion and disadvantage. Nonetheless, we have argued here that health systems should be understood as social determinants themselves, which can function as spaces to mitigate exclusion and weave together fragmented and deeply polarized societies. A fundamental role for such a social institution is a democratically legitimate as well as scientifically valid process for defining the contours of claimable entitlements, which is then subsequently regulated. Thus, democratically legitimate priority-setting should be seen as part of designing a health system based upon the right to health, not in conflict with it. Such processes should be explicit, recognizing that implicit priority-setting does not provide transparency or morally acceptable justification, and incorporate meaningful citizen participation.

This analysis points to the need for dialogue between judicial and legal actors, and health policymakers in the region relating to the importance of and their potential roles in enhancing fair and legitimate priority-setting processes. Further, it underscores the importance of improved and more actionable information to better understand, and in turn effectively address, the dynamics between priority-setting, regulation and judicial enforcement of entitlements within and across contexts.

## Data Availability

Not applicable

## Abbreviations

UHC: Universal Health Coverage
WHO: World Health Organization

## References

[1] World Health Organization (2008). Closing the gap in a generation: commission on social determinants of health final report.

[2] Petter Ottersen O, Dasgupta J, Blouin C, Buss P, Virasakdi C, Frenk J (2014). The political origins of health inequity: prospects for change. Lancet..

[3] Bossert T, Blanchet N, Sheetz S, Pinto D, Cali J, Cuevas Pérez R, Comparative review of health system integration in selected countries in Latin America, Inter-American Development Bank [IDB] technical note IDB-TN-585. 2014 Jan. Available from: https://publications.iadb.org/bitstream/handle/11319/6024/Technical%20Note%20585-%20Health%20System%20Fragmentation.pdf.

[4] Rumbold B, Baker R, Ferraz O, Hawkes S, Krubiner C, Littlejohns P (2017). Universal health coverage, priority setting, and the human right to health. Lancet..

[5] Yamin AE. The right to health in Latin America: the challenges of constructing fair limits. Penn J of Intl law. 2019;40(3):695–732.

[6] Daniels N (2008). Just health: meeting health needs fairly.

[7] Baltussen R, Jansen M P, Bijlmakers L, Tromp N, Yamin A E, Norheim O F (2017). Progressive realisation of universal health coverage: what are the required processes and evidence?. BMJ Global Health.

[8] Charvel S, Cobo F, Larrea S, Baglietto J (2018). Challenges in priority setting from a legal perspective in Brazil, Costa Rica, Chile, and Mexico. Health and hum. Rights..

[9] WHO Consultative Group on Equity and Universal Health Coverage. Making fair choices on the path to universal health coverage. Geneva: World Health Organization; 2014.

[10] Pichon-Riviere A, GarciaMarti S, Oortwijn W, Augustovski F, SampietroColom L (2019). Defining the value of health Technologies in Latin America: developments in value frameworks to inform the allocation of healthcare resources. Int J Technol Assess Health Care.

[11] Syrett K (2007). Law, legitimacy, and the rationing of health care: a contextual and comparative perspective.

[12] Ferraz OLM (2018). Health in the courts of Latin America. Health and Hum Rights.

[13] Rodriguez-Garavito C (2011). Beyond the courtroom: the impact of judicial activism on socioeconomic rights in Latin America. Tex L Rev.

[14] Bergallo P, Yamin AE, Gloppen S (2011). Argentina: courts and the right to health: achieving fairness despite “routinization” in individual coverage cases?. Litigating health rights: can courts bring more justice to health?.

[15] Biehl J, Socal MP, Gauri V, Diniz D, Medeiros M, Rondon G, Amon JJ (2019). Judicialization 2.0: understanding right-to-health litigation in real time. Glob Public Health.

